# Metagenomic Comparison of Gut Microbes of *Lemur catta* in Captive and Semi-Free-Range Environments

**DOI:** 10.3390/ani15101442

**Published:** 2025-05-16

**Authors:** Chunzhong Xu, Xinzi Guo, Lian Li

**Affiliations:** 1 Shanghai Wild Animal Park, Shanghai 201399, China; xucz70@126.com; 2 College of Animal Science and Technology, Nanjing Agricultural University, Nanjing 210095, China; 15121225@stu.njau.edu.cn; 3 MOE Joint International Research Laboratory of Animal Health and Food Safety, College of Veterinary Medicine, Nanjing Agricultural University, Nanjing 210095, China

**Keywords:** *Lemur catta*, metagenomic analysis, microbiome, microbiota function, CAZymes

## Abstract

In order to protect endangered species, many zoos adopt diverse rearing models to achieve optimal conservation outcomes. This study employed metagenomic approaches to assess differences in the fecal microbiome of captive and semi-free-ranging ring-tailed lemurs (*Lemur catta*). The results show that captivity significantly altered the microbial community structure. The inter-individual variability in the microbial community within the captive-bred (CB) group was lower than that in the semi-free-ranging (FR) group, yet these individuals harbored a higher abundance of potential pathogens (*Treponema_D*). In contrast, microbial genera associated with fiber degradation and short-chain fatty acid production in the FR group were significantly elevated (*Faecalibacterium*, *Roseburia*, and *Megamonas*) as compared to the CB group. Environmental variations between the two rearing systems led to distinct profiles in microbial functions and carbohydrate-active enzyme gene composition. Notably, the FR group of lemurs exhibited an increased abundance of enzyme genes associated with the degradation of complex polysaccharides (cellulose, hemicellulose, and pectin), suggesting that their diet, rich in natural plant fibers, enhances the capacity of their gut microbiota to extract essential energy and nutrients. Conversely, the CB group displayed a more homogeneous microbial community with a higher prevalence of potential pathogens, implying that a captive lifestyle may negatively impact gastrointestinal health. These findings offer valuable insights into the influence of rearing conditions on gut microbial ecology and its potential implications for the health management of ring-tailed lemurs.

## Simple Summary

This study employed metagenomic methods to assess the differences in the fecal microbiomes of captive and semi-free-ranging ring-tailed lemurs (*Lemur catta*). The results indicate that captivity alters the community structure of the fecal microbiota of *Lemur catta*. Differences in the living environment resulted in variations in microbial functions and in the profiles of carbohydrate-active enzyme genes. Furthermore, the microbial community composition among captive ring-tailed lemurs was more homogeneous, yet it harbored a higher abundance of potential pathogens, suggesting that a captive lifestyle may adversely affect gastrointestinal health.

## 1. Introduction

Functioning as a crucial regulatory system for host health, the gut microbiota exhibits compositional and functional characteristics highly responsive to dietary patterns, environmental exposures, and host behaviors [1,2,3,4,5]. Significant alterations in intestinal microbial communities frequently occur when wild animals transition between natural habitats and captive environments, potentially influencing metabolic processes, immune function, and disease susceptibility [6]. Endemic to Madagascar with vulnerable conservation status, ring-tailed lemurs (*Lemur catta*) are bred in most zoos across China, with Shanghai Wild Animal Park housing approximate 300 individuals, forming the largest population in the country. In addition, ring-tailed lemurs represent an ideal model system for investigating associations between microbiome composition and species survival research, holding considerable value for endangered species conservation [7,8]. Despite their importance, systematic investigations into differential gut microbial compositions and functional adaptation mechanisms across various management regimes in *Lemur catta* remain limited, with few studies providing comparative data and an even greater lack of functional metagenomic analyses to elucidate microbial roles in host adaptation [9]. Furthermore, previous studies have primarily focused on lemur populations in their native range or in Western zoos, with limited attention given to large-scale populations under managed care in China. Consequently, little is known about how different captive management strategies implemented within Chinese facilities impact gut microbial ecology and functional adaptation in ring-tailed lemurs [10]. This study addresses this knowledge gap by conducting a high-resolution metagenomic comparison of captive and free-ranging individuals in one of China’s largest managed lemur populations.

In the context of China’s rapidly expanding ex situ conservation programs, understanding how local management conditions affect host-associated microbiomes is essential for improving animal welfare and health surveillance [11]. Comparison between enclosure-restricted versus semi-free-ranging management strategies provides a natural experimental paradigm for exploring environment–host–microbiome interactions [12]. Under captive conditions, individuals are isolated from visitors and rely exclusively on provisioned diets, which likely leads to microbial communities that differ substantially from those of conspecifics managed under semi-free-ranging conditions [9]. Shotgun metagenomic analyses of captive lemurs with different feeding strategies have demonstrated significant differences in microbial community composition and functional potential, indicating that diet and management conditions jointly shape the gut microbiome and its ecological functions [13]. Divergent food sources between enclosure-restricted and semi-free-ranging management not only influence intestinal microbial diversity in *Lemur catta* but may also significantly affect the dominant microbial communities and their abundance [9]. These dietary differences extend beyond mere nutritional composition to encompass physical properties as well. For instance, variations in food texture and hardness can impact dental wear and tooth loss in these primates, potentially creating secondary effects on digestive efficiency and microbiome composition [14,15,16]. Furthermore, semi-free-ranging individuals may acquire more complex microbial colonization patterns and metabolic substrates through visitor interactions and the potential consumption of non-provisioned foods (either through tourist offerings or environmental resources) [17]. Beyond microbial diversity metrics, such distinctions potentially manifest in functional gene distribution, affecting critical physiological processes, including nutritional metabolism and pathogen defense [18,19,20]. Comprehensive resolution of microbial community taxonomic composition and functional potential comes through metagenomic sequencing technology, offering high-resolution data for elucidating the mechanisms underlying environmental pressures and host adaptation [21].

Employing a metagenomic approach, this study aimed to elucidate structural differences in gut microbial communities between captive cred and semi-free-ranging ring-tailed lemurs and how environmental exposure through visitor contact and dietary diversity influence microbial functional pathways. The results from our study are expected to provide a scientific foundation for optimizing captive management strategies and assessing the ecological risks associated with semi-free-ranging models, while expanding the understanding of primate–microbiome symbiotic relationships in artificial environments.

## 2. Materials and Methods

### 2.1. Animals and Housing Conditions

**Figure 1:**
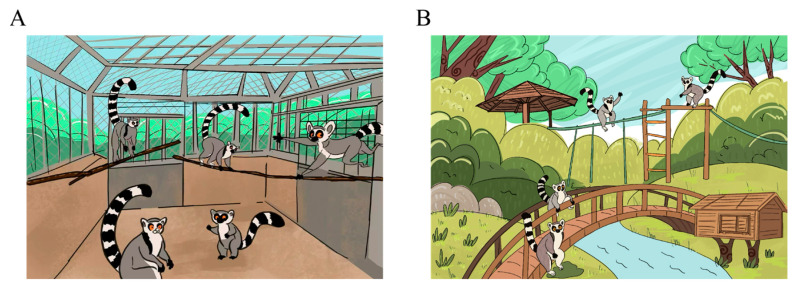
Comparison of environmental conditions for ring-tailed lemurs (*Lemur catta*) in different housing systems. (**A**) Captive environment: sixteen lemurs were housed in two indoor–outdoor enclosures (eight per enclosure), each approximately 23 m^2^ in size. (**B**) Semi-free-range environment: approximately 120 lemurs were kept on a 10,000 m^2^ vegetated island (green coverage ~3000 m^2^) equipped with shelters such as pavilions and wooden cabins.

In this study, ring-tailed lemurs were maintained under two distinct rearing systems within the same facility, enabling comparisons of gut microbiota between captive-bred (CB) and semi-free-ranging (FR) populations. The FR lemurs were housed on an artificial island measuring approximately 10,000 m^2^, surrounded by water. The island environment included around 3000 m^2^ of vegetated areas, pavilions, and heated wooden shelters. It supported a population of about 120 ring-tailed lemurs, including 50 juveniles born in 2024, all free to move and interact. The animals had access to natural vegetation and engaged in routine direct contact with tourists, resulting in high levels of human exposure and environmental complexity. The CB lemurs were housed in enclosure systems, each accommodating eight individuals. Each system comprised indoor (approximately 23 m^2^, 2.8 m high) and outdoor (approximately 23 m^2^, 2.1 m high) spaces, allowing for free movement between them (Figure 1). During winter, oil radiators were used to maintain indoor temperatures above 10 °C, enabling thermoregulatory behavior. In contrast to the FR group, the CB animals were completely isolated from visitors. They received a standardized, human-managed diet primarily composed of steamed cornbread, seasonal fruits and vegetables (e.g., apples, bananas, cucumbers, carrots), red dates, mealworms, and weekly portions of nuts. Multivitamins, trace elements, and milk powder were also added regularly. All individuals in both groups weighed between 1.8 and 3.0 kg and had reached or were approaching sexual maturity, typically between 2 and 3 years of age in this species.

### 2.2. Dietary Management

Both management groups received identical diets to control for nutritional variables, though FR lemurs potentially had access to supplemental items from visitors despite regulations prohibiting such feeding [17]. The standardized diet consisted of a staple carbohydrate base (steamed corn bread), seasonal fresh fruits (apples, bananas, pears, oranges), seasonal vegetables (cucumbers, carrots, various leafy greens), supplemental items (dates, mealworms), a weekly provision of measured quantities of nuts and dried fruits, and regular nutritional supplementation with multivitamins, trace minerals, and milk powder. This dietary regimen was designed to approximate the nutritional profile of wild lemur diets while ensuring complete nutrition in captivity [19]. While CB lemurs received only these provisioned items, semi-free-ranging individuals potentially accessed additional food sources through visitor interactions or environmental foraging, representing a key variable potentially influencing microbial community composition.

### 2.3. Sample Selection Criteria and Sample Collection

Inclusion criteria for fecal samples were as follows: (1) Healthy adult individuals with no antibiotic treatment in the past six months. (2) Comparable age range (3–4 years). (3) Stable diet for at least one year prior to sampling. Exclusion criteria: Any individual exhibiting gastrointestinal symptoms or having received medical interventions within six months of sampling.

Fourteen fecal samples were collected from 3-to-4-years-old animals in each management group. For CB lemurs, samples were collected during morning cleaning procedures to ensure freshness within 6 h post-defecation. For FR lemurs, animals were observed from a distance, and fresh fecal samples were collected immediately following observed defecation events to establish sample identity and freshness. All samples were collected using sterile techniques with DNA/RNA-free collection materials. Approximately 2–3 g of fecal material was collected per sample, with care taken to avoid environmental contamination. Samples were immediately frozen in liquid nitrogen and transported to the laboratory where they were stored at −80 °C until DNA extraction. Inclusion criteria: (1) Healthy adult individuals with no antibiotic treatment in the past six months. (2) Comparable age range (3–4 years). (3) Stable diet for at least one year prior to sampling. Exclusion criteria: Any individual exhibiting gastrointestinal symptoms or having received medical interventions within six months of sampling.

### 2.4. DNA Extraction and Metagenomic Sequencing

Microbial DNA extraction and shotgun metagenomic sequencing were conducted following a standardized protocol adapted for non-human primate samples [9]. Fecal genomic DNA was isolated using a commercial extraction kit optimized for primate feces. DNA purity and integrity were evaluated using a NanoDrop spectrophotometer, and only samples with an A260/A280 ratio between 1.8 and 2.0 were used for downstream applications. Sequencing libraries were prepared from 200 ng of high-quality DNA using standard protocols and were subjected to rigorous quality control before sequencing. Libraries were sequenced on the Illumina HiSeq X platform (Illumina, San Diego, CA, USA), generating 2 × 150 bp paired-end reads. Each sample yielded more than 20 million quality-filtered reads to ensure a sufficient sequencing depth for gut microbiome analysis.

### 2.5. Bioinformatic Analysis

Raw sequencing data were quality-trimmed using Trimmomatic (v0.36) to remove low-quality reads and adapter sequences. Host-derived reads were filtered out by aligning to the Lemur catta reference genome using BWA (v0.7.17). Clean reads were de novo assembled into contigs using MEGAHIT (v1.1.2) with a minimum contig length of 500 bp. Open reading frames (ORFs) were predicted using Prodigal (v2.6.3), and a non-redundant gene catalog was generated using CD-HIT (v4.6.7). Functional annotation was performed by aligning the predicted protein sequences against the KEGG (v90.0), eggNOG, and CAZy databases using DIAMOND (v2.0.11.149, https://github.com/bbuchfink/diamond, accessed on 12 May 2025).

Taxonomic profiling was performed using MetaPhlAn3 (v3.0.14, https://github.com/biobakery/metaphlan, accessed on 12 May 2025), and community composition was analyzed at the domain, phylum, genus, and species levels. Microbial diversity was evaluated using both alpha diversity indices (Richness, Shannon, Chao1, Faith’s Phylogenetic Diversity) and beta diversity measures (Bray–Curtis dissimilarity, weighted and unweighted UniFrac distances). Linear discriminant analysis (LDA) effect size (LEfSe) was used to identify differentially abundant taxa and functional features (LDA > 3, *p* < 0.05). Differential abundance analysis of microbial taxa, functional genes (KEGG), and carbohydrate-active enzyme (CAZyme) genes was conducted using DESeq2 (v1.38.3, https://bioconductor.org/packages/DESeq2, accessed on 12 May 2025) with compositional data transformation. Antimicrobial resistance genes (ARGs) were identified and categorized based on type, mechanism, and rank.

### 2.6. Statistical Analysis

All statistical analyses were performed in R (v4.2.0) using specialized packages for microbiome research. Data normality was tested with the Shapiro–Wilk test. For normally distributed data, differences between groups were evaluated using one-way ANOVA followed by Duncan’s post hoc test. For non-normally distributed data, the Kruskal–Wallis test was applied. Alpha diversity indices were compared using non-parametric tests with Benjamini–Hochberg correction for multiple comparisons. Beta diversity was visualized using principal coordinate analysis (PCoA) and assessed statistically using permutational multivariate analysis of variance (PERMANOVA). Spearman’s rank correlation coefficient was used to explore associations between microbial features and environmental variables. Statistical significance was accepted at *p* ≤ 0.05, while results with 0.05 < *p* ≤ 0.10 were interpreted as indicative of a statistical trend rather than definitive significance.

## 3. Results

### 3.1. The Fecal Microbiota Composition at Domain Level and Viral Level

**Figure 2:**
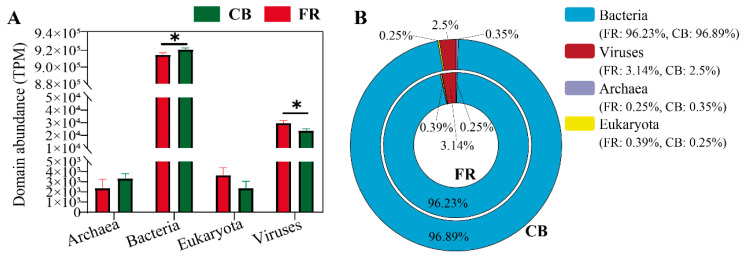
Relative abundance of fecal microbiota at the domain level in *Lemur catta*. (**A**) Relative abundance of fecal microbiota at domain level among the groups. (**B**) Domain-level percentage distribution of fecal microbiota. CB: captive-bred group; FR: semi-free-ranging group. The data are presented as means ± SEM (n = 14). Asterisks (*) represent significant differences with *p* < 0.05 and *p* < 0.01. Note: Percentages may not total exactly 100% due to rounding.

The relative abundance of the fecal microbiota at the domain level was first assessed between two groups. As shown in Figure 2A, the bacterial abundance in the CB group was significantly higher than in the FR group, while the viral abundance was significantly lower (*p* < 0.05). Figure 2B illustrates the relative abundance of bacteria, archaea, fungi, and viruses across groups, with bacteria accounting for the dominant proportion, exceeding 95%.

**Figure 3:**
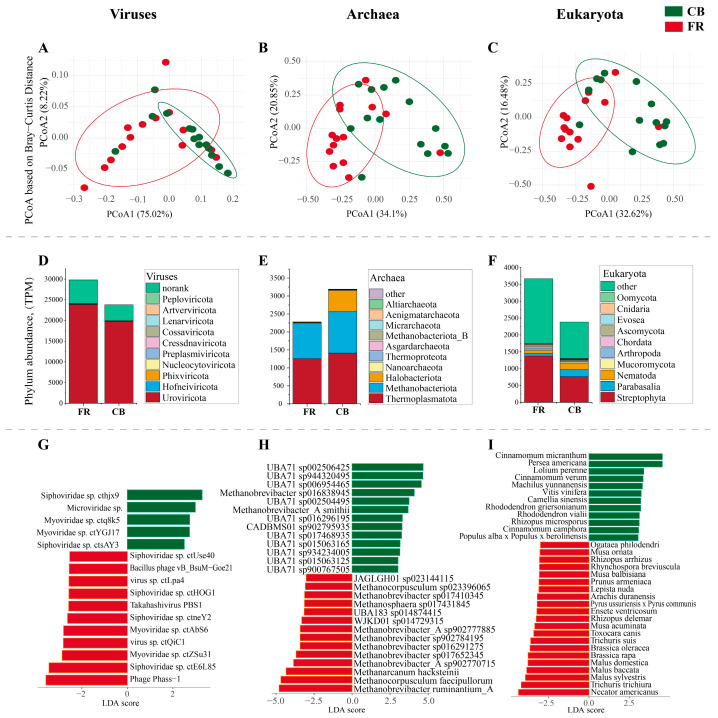
Relative abundance of fecal microbiota at the viral, archaea, and eukaryota levels in *Lemur catta*. (**A**–**C**) Principal coordinate analysis based on the Bray–Curtis distance at viral level between the CB and FR groups. (**D**–**F**) Top 10 phylum in the two groups. (**G**–**I**) Linear discriminant analysis effect size of fecal microbiota at species level (LDA > 3, *p*  <  0.05). CB: captive-bred group; FR: semi-free-ranging group. The data are presented as means ± SEM (n = 14).

Subsequent compositional analysis at the viral, archaea, and eukaryota level using principal coordinate analysis (PCoA) revealed significant differences between the two groups (*p* < 0.01, Figure 3A–C). Figure 3D presents the top 10 viral phyla, and Uroviricota and Hofneiviricota were the dominant phyla. Figure 3E presents the top 10 phyla at the archaea level, with Thermoplasmatota, Methanobacteriota and Halobacteriota as the dominant phyla. Figure 3F presents the top 10 phylum at the eukaryota level, and Streptophyta, Parabasalia and Nematoda were the dominant phyla. Five species of viruses, including *Siphoviridae* sp. *cthjx9* and *Microviridae* sp., exhibited increased abundance in the FR group, whereas 11 species of viruses, including *Phage Phass-1* and *Siphoviridae* sp. *ctE6L85*, showed a significant increase in the CB group (LDA > 3, *p* < 0.05, Figure 3G). Thirteen species of archaea, including *Methanobrevibacter ruminantium_A* and *Methanocorpusculum faceipullorum*, exhibited increased abundance in the FR group, whereas 13 species of viruses, including *Methanobrevibacter_A smithii* and *Methanobrevibacter sp016838945*, showed a significant increase in the CB group (LDA > 3, *p* < 0.05, Figure 3H). Twenty-one species of archaea, including *Necator americanus*, *Trichuris trichiura* and *Malus sylvestris* exhibited increased abundance in the FR group, whereas 13 species of viruses, including *Cinnamomum micranthum* and *Persea americana*, showed a significant increase in the CB group (LDA > 3, *p* < 0.05, Figure 3I).

### 3.2. Analysis of Fecal Microbial Diversity and Composition at Bacterial Level

**Figure 4:**
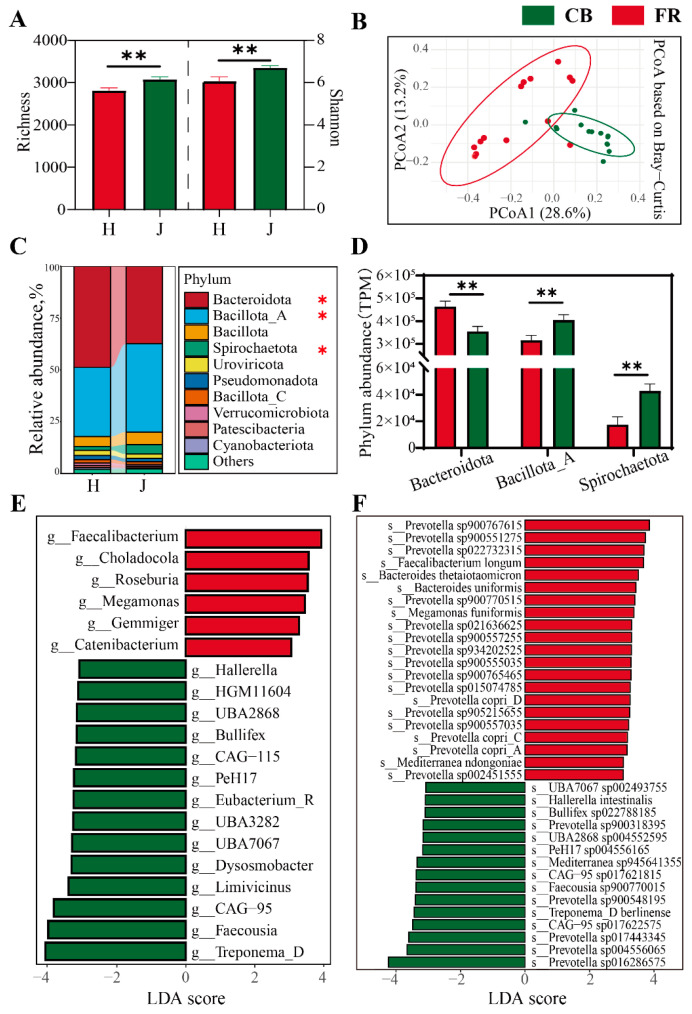
Analysis of fecal microbial diversity and composition at the bacterial level. (**A**) Richness and Shannon indexes of the two groups. (**B**) The principal coordinate analysis plots based on Bray–Curtis distance among the groups. (**C**) The relative abundance of microbial composition at the phylum level. (**D**) The core phylum with significant differences between the two groups. (**E**) Linear discriminant analysis effect size of fecal microbiota at genus level (LDA > 3, *p* < 0.05). (**F**) Linear discriminant analysis effect size of fecal microbiota at species level (LDA > 3, *p*  < 0.05). CB: captive-bred group; FR: semi-free-ranging group. The data are presented as means ± SEM (n = 14). Asterisks (* and **) represent significant differences with *p* < 0.05 and *p* < 0.01.

The composition and diversity of fecal microbiota at the bacterial level was assessed. Compared to the FR group, the α-diversity of fecal microbiota significantly increased in the CB group, evidenced by the increased richness index and Chao1 index (*p* < 0.05, Figure 4A). PCoA revealed significant differences between the two groups (*p* < 0.01, Figure 4B). The phyla Bacteroidota, Bacillota_A, Bacillota, and Spirochaetota were dominant (*p* < 0.05, Figure 4C). Interestingly, the relative abundance of Bacteroidota was significantly increased in the FR group, while the relative abundances of Bacillota_A and Spirochaetota were significantly increased in the CB group (Figure 4D). Figure 4E,F shows the linear discriminant analysis effect size (LEfSe) of the fecal microbiota at the genus level and species level. At the genus level, the FR group showed significantly higher relative abundances of *Faecalibacterium*, *Roseburia*, *Gemmiger*, and *Catenibacterium*, which are key producers of short-chain fatty acids. The CB group showed significantly higher relative abundances of *Treponema_D*, *Faecousia*, *Hallerella,* and 14 other genera (LDA > 3, *p* < 0.05). At the species level, the FR group showed significantly higher relative abundances of *Faecalibacterium longum*, *Bacteroides thetaiotaomicron*, *Bacteroides uniformis*, *Prevotella copri_A*, *C* and *D*, and 15 other species. The CB group showed significantly higher relative abundances of *Hallerella intestinalis*, *Treponema_D berlinense,* and 13 other species. Notably, both groups showed a significant enrichment of *Prevotella* species: 16 species were significantly upregulated in the FR group versus 5 in the CB group.

### 3.3. Metagenomic Analysis of the Differences in Metabolic Pathways

**Figure 5:**
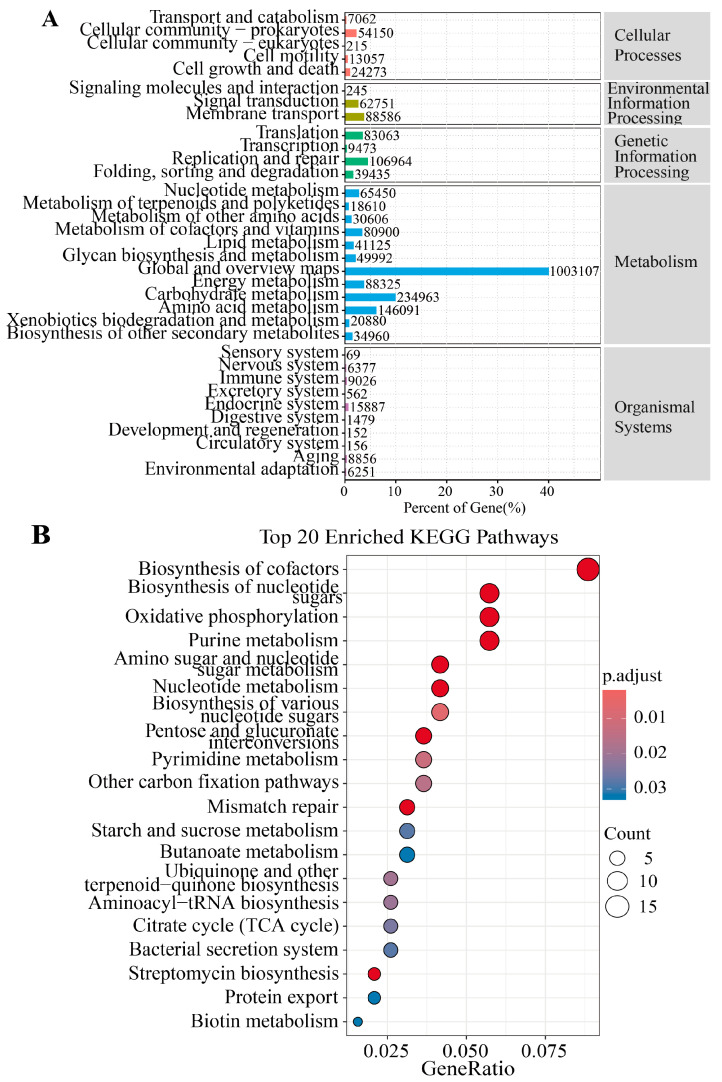
Metagenomic analysis of the differences in metabolic pathways. (**A**) KEGG annotation statistics plot. The *y*-axis displays the names of the KEGG metabolic pathways, and the *x*-axis represents both the number of genes annotated to each pathway and the percentage that these genes constitute the total annotated gene pool. (**B**) Top 20 KEGG pathways enriched in differentially expressed genes (DEGs) between the two groups. The data are presented as means ± SEM (n = 14).

**Figure 6:**
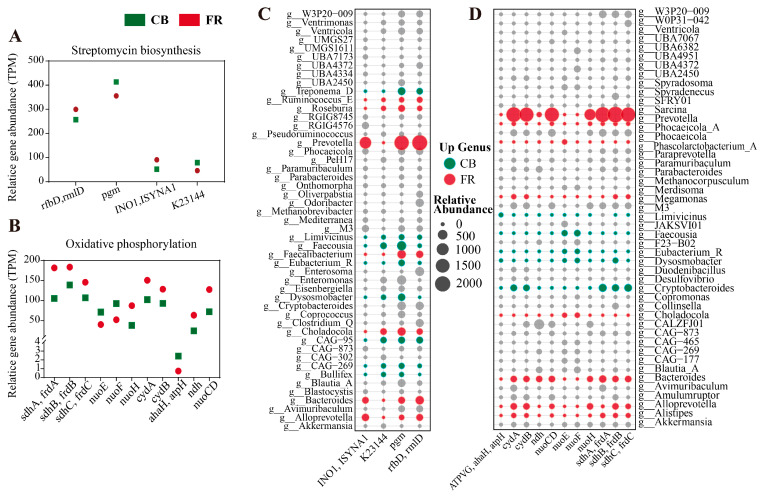
Metagenomic analysis of the differences in metabolic pathways. (**A**) DEGs mapped to the streptomycin biosynthesis pathway. (**B**) DEGs mapped to the oxidative phosphorylation pathway. (**C**) Genera contribution profile to differential genes (streptomycin biosynthesis). (**D**) Genera contribution profile to differential genes (oxidative phosphorylation). The red or green denotes the differential genera that contribute genes. CB: captive-bred group; FR: semi-free-ranging group. The data are presented as means ± SEM (n = 14).

Figure 5A illustrates the proportional distribution of genes across major KEGG functional categories in the FR and CB groups. Pathways related to metabolism and genetic information processing dominated, collectively accounting for the highest gene abundance. Figure 5B presents the top 20 KEGG pathways enriched by differential genes, and pathways such as “Biosynthesis of cofactors”, “Streptomycin biosynthesis” and “Oxidative phosphorylation” were significantly enriched (*p* < 0.05). Subsequently, the differential genes in the streptomycin biosynthesis and oxidative phosphorylation pathways were characterized (*p* < 0.05, Figure 6C,D). In the pathway of streptomycin biosynthesis, the relative abundances of *rfbD* (*rmlD*) and *INO1* (*ISYNA1*) were significantly higher in the FR group, while the relative abundances of *pgm* and *K23144* were significantly higher in the CB group (*p* < 0.05, Figure 6C). In the pathway of oxidative phosphorylation, the relative abundances of *sdhC* (*frdC*), *sdhB* (*frdB*), *sdhA* (*frdA*), *cydB*, *cydA*, *nuoH*, *nuoCD,* and *ndh* were significantly higher in FR group, while the relative abundances of *nuoE*, *nuoF,* and *ahaH*(*atpH*) were significantly higher in the CB group.

Subsequently, the differential genes in the streptomycin biosynthesis and oxidative phosphorylation pathways were characterized (*p* < 0.05, Figure 6A,B). In the pathway of streptomycin biosynthesis, the relative abundances of *rfbD* (*rmlD*) and *INO1* (*ISYNA1*) were significantly higher in FR group, while the relative abundances of *pgm* and *K23144* were significantly higher in CB group (*p* < 0.05, Figure 6A). In the oxidative phosphorylation pathway, the relative abundances of *sdhC* (*frdC*), *sdhB* (*frdB*), *sdhA* (*frdA*), *cydB*, *cydA*, *nuoH*, *nuoCD* and *ndh* were significantly higher in FR group, while the relative abundances of *nuoE*, *nuoF* and *ahaH*(*atpH*) were significantly higher in the CB group (*p* < 0.05, Figure 6B). As shown in Figure 6C, the significantly increased abundance of the *pgm* gene in the CB group was primarily associated with *Treponema_D*, *Faecousia*, and *Dysosmobacter*. The significantly increased abundance of the *K23144* gene was mainly linked to *Faecousia* and *CAG-95*. In the FR group, the significantly elevated abundance of the *rfbD* and *INO1* genes was predominantly associated with *Prevotella*, *Bacteroides*, and *Alloprevotella*. As illustrated in Figure 6D, the significantly increased abundance of the *cydA*, *cydB*, *ndh*, *nuoCD*, *nuoH*, *sdhA*, *sdhB*, and *sdhC* genes in the FR group was mainly linked to *Prevotella*, *Bacteroides*, *Alistipes*, and *Alloprevotella*. In the CB group, the significantly elevated abundance of the *nuoE* and *nuoF* genes was predominantly associated with *Faecousia*, *Eubacterium_R*, and *Dysosmobacter*, while the significantly increased abundance of the *atpH* gene was mainly linked to *Liimivicinus* and *Dysosmobacter*.

### 3.4. Metagenomic Analysis of the Differentially Represented Genes Encoding CAZymes

**Figure 7:**
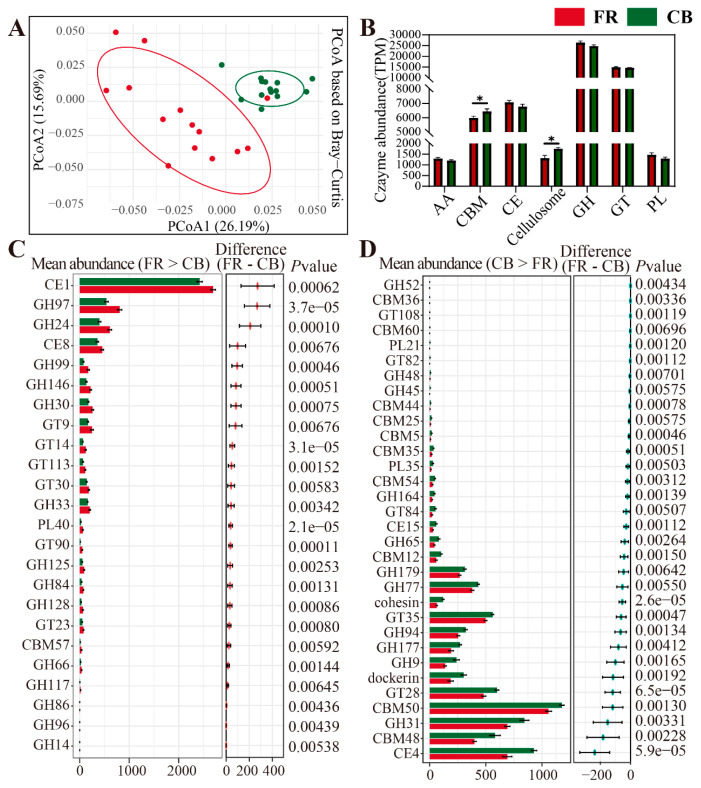
Metagenomic analysis of the differentially represented genes encoding CAZymes. (**A**) The differences in CAZyme gene profiles between the CB and FR groups based on the principal coordinate analysis using Bray–Curtis distances. (**B**) The relative abundance of microbial Cazymes composition at the class level. (**C**) The statistically significant CAZymes in the two groups (FR higher than CB). (**D**) The statistically significant CAZymes in the two groups (CB higher than FR). CB: captive-bred group; FR: semi-free-ranging group. The data are presented as means ± SEM (n = 14). Asterisks (*) represent significant differences with *p* < 0.05 and *p* < 0.01.

Due to the differences in environments and diets between CB and FR groups, their capacities for carbohydrate metabolism may also differ. Therefore, we assessed the differential CAZymes in the two groups. PCoA revealed significant differences between the two groups (*p* < 0.01, Figure 7A). The relative abundance of the microbial CAZymes composition at the class level is shown in Figure 7B, and the relative abundances of CBM and Cellulosome were significantly higher in the CB group (*p* < 0.05). The relative abundances of AA, CE, GH, GT, and PL were higher in the FR group, but there was no statistical significance (*p* > 0.05). Next, the CAZymes with significant differences at the family level between the two groups were characterized. In the FR group, 24 CAZymes were significantly higher than those in the CB group, with the top five differential enzymes were CE1, GH97, GH24, CE8, and GH99. In the CB group, 32 CAZymes were significantly higher than those in the FR group, and the top five differential enzymes were CE4, CBM48, GH31, CBM50, and GT28.

### 3.5. Metagenomic Analysis of Antimicrobial Resistance Genes (ARGs)

**Figure 8:**
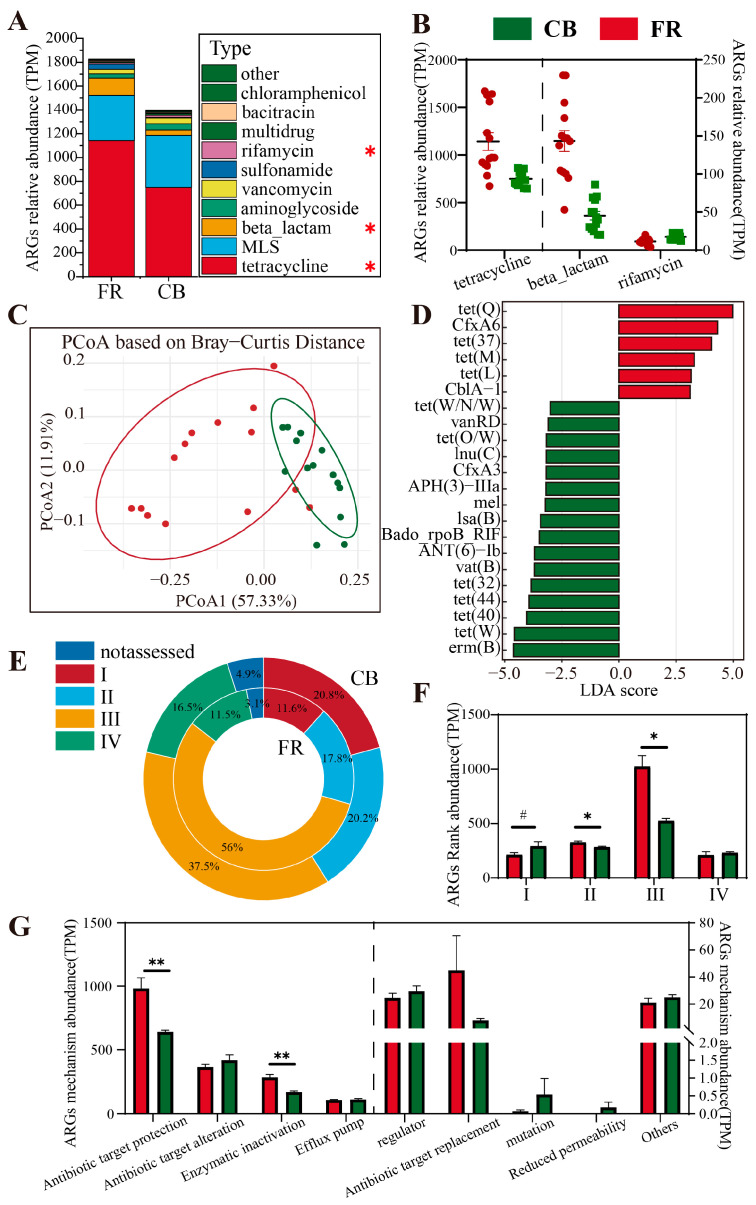
Metagenomic analysis of Antimicrobial Resistance Gene (ARG) differences. (**A**) The relative abundance of ARG composition at the type level. (**B**) The core type with significant differences between the two groups. (**C**) The principal coordinate analysis plots based on Bray–Curtis distance among the groups. (**D**) Linear discriminant analysis effect size of ARGs at the subtype level (LDA > 3, *p* < 0.05). (**E**) Rank level percentage distribution of ARGs. (**F**) The relative abundance of ARG composition at the rank level. (**G**) The relative abundance of ARG composition at the mechanism level. CB: captive-bred group; FR: semi-free-ranging group. The data are presented as means ± SEM (n = 14). Asterisks (#) represent differences with *p* < 0.1. Asterisks (* and **) represent significant differences with *p* < 0.05 and *p* < 0.01. Note: Percentages may not total exactly 100% due to rounding.

The analysis of antibiotic resistance genes at the type level for the two groups revealed that the dominant phyla were tetracycline, MLS, and beta-lactam (*p* < 0.05, Figure 8A). In the FR group, the abundances of tetracycline and beta-lactam were significantly higher than those in the CB group, whereas the abundance of rifamycin was significantly lower (*p* < 0.05, Figure 8B). Beta diversity analysis demonstrated significant differences in ARGs between the two groups (*p* < 0.01, Figure 8C). Subsequently, LEfSe analysis at the subtype level showed that six subtypes of ARGs (including *tet(Q)* and *CfxA6*) were significantly higher in the FR group compared to the CB group, while 16 subtypes of ARGs (including *erm(B)* and *tet(W)*) were significantly higher in the CB group compared to the FR group (LDA > 3, *p* < 0.05, Figure 8D). At the rank level, rank 1 exhibited a significant upward trend in the CB group (*p* < 0.1, Figure 8F), whereas ranks 2 and 3 were significantly higher in the FR group relative to the CB group (*p* < 0.05, Figure 8E,F). Finally, at the mechanism level, the evaluation of antibiotic gene abundance indicated that antibiotic target protection and enzymatic inactivation in the FR group were significantly higher than in the CB group (*p* < 0.05, Figure 8G).

## 4. Discussion

Through metagenomic analysis of the fecal microbiota of ring-tailed lemurs under different husbandry conditions, we found that captivity alters the community structure of the fecal microbiota in these animals. Moreover, significant differences in microbial functions and carbohydrate-active enzyme genes were observed between the captive and semi-free-ranging groups. The similar environmental conditions experienced in captivity result in reduced inter-individual variation in the intestinal microbiota among captive ring-tailed lemurs. In addition, captive lemurs had more potential pathogens, which could cause gastrointestinal problems, indicating that captive life might affect the gastrointestinal health of ring-tailed lemurs.

Viruses that infect bacteria (phages) are the most abundant biological entities on this planet [22]. Captive and semi-free-ranging environments harbor significantly distinct viral communities, which results in significant differences in the viral structures within the microbiota of the two groups of ring-tailed lemurs. Consequently, differences in the structures and abundances of bacteria also emerge [23]. Given that most bacteriophages exhibit high host specificity, even modest shifts in bacterial community structure—driven by diet, environmental exposures, or host-related factors—can lead to rapid virome restructuring. Therefore, the distinct viral profiles observed between the two groups may reflect altered phage–host dynamics resulting from captivity-induced microbiota changes [24,25]. Archaea are single-celled organisms with unique features that form one of the three major domains of life, alongside bacteria and eukarya. Initially believed to exist only in harsh environments such as hot springs, they are now recognized as key members of the human microbiome [26]. These microorganisms play essential roles in intestinal ecological balance, nutrient metabolism, and greenhouse gas (methane) emissions. In human feces, the most prevalent (>90%) archaeal representatives are *Methanobrevibacter smithii* and *Candidatus Methanobrevibacter intestine* [27,28]; in ruminants, *Methanobrevibacter ruminantium* has been detected [29]. In our study, differences in *Methanobrevibacter* were detected in both groups. In the FR group, the abundance of *Methanobrevibacter ruminantium* was significantly increased. This species has been reported to be capable of degrading various lignocelluloses, suggesting that ring-tailed lemurs in the FR group may ingest and break down more lignocellulose [30,31]. Semi-free-ranging environments may enable animals to access a more diverse diet with greater fiber and forage availability. In contrast, the CB group exhibited a significant enrichment of *Methanobrevibacter smithii*, a bacterium that has been reported to be potentially associated with inflammatory responses [32,33]. This finding implies that a captive environment might lead to intestinal inflammation in ring-tailed lemurs. Fungi in the gut may play both probiotic and potentially pathogenic roles; they are capable of interacting with bacteria, viruses, and other microorganisms, thereby influencing the host’s immune response [34,35,36]. Variations in fungal communities under different rearing practices may affect animal gut health, nutrient absorption, and disease susceptibility [37]. In our study, in the CB group, a significant increase in the abundance of fungi such as *Persea americana* and *Cinnamomum verum* was observed. These fungi have been reported to enhance anti-inflammatory responses or exert antioxidant effects [38,39,40,41]. In the FR group, a significant enrichment of fungi such as *Necator americanus* and *Trichuris trichiura* was observed. This suggests that increased exposure to soil, vegetation, and external microorganisms in a natural environment may lead to a fungal community with higher diversity.

Previous studies have demonstrated that captive breeding significantly modified the daily behaviors of ring-tailed lemurs, including stress-related activities, movement, and social interactions [7]. Furthermore, changes in the environment significantly affected the physiology and health of ring-tailed lemurs and resulted in notable alterations in both the structure and abundance of their gut microbiota [5,12]. In our study, the dominant phyla in both captive-bred and semi-free-ranging golden lemurs were Bacteroidetes and Bacillota_A (Figure 4C), consistent with findings from previous studies on primate gut microbiota [42]. The CB group exhibited a significantly higher alpha diversity compared to the FR group, aligning with established research findings. In beta-diversity analysis, the microbial composition within the CB group demonstrated reduced inter-individual variation compared to that in the FR group. Previous research has documented a trend toward human-like microbiome composition in captive animals [43,44]. Both phenomena may result from the relatively homogeneous environment in captivity, driving changes in intestinal microbiota structure [45,46]. Although ring-tailed lemurs in the FR group may acquire additional food items (fruits and bread) through contact with tourists, the daily diets of the two groups (fruits, vegetables, milk powder, and bread) are essentially similar. We speculate that this phenomenon arises from the markedly different environments in which the two groups reside. The enclosed environment minimizes interference from exogenous microorganisms, allowing the bacterial communities to reach higher abundances under reduced competitive pressures and to exhibit a more homogeneous composition. In contrast, the FR group lemurs, which engage in activities in the wild, inhabit relatively complex environments where competitive pressures among bacterial communities are greater, resulting in considerable fluctuations in the composition and abundance of fecal microbiota among different individuals within the FR group. LDA at the genus level revealed that *Faecalibacterium*, *Roseburia*, and *Gemmiger* were significantly increased in the FR group. These genera have been consistently reported in prior studies as key short-chain fatty acid (SCFA) producers [47,48,49,50]. At the species level, the FR group exhibited a significant enrichment of multiple *Prevotella*-related populations, which are typically associated with diets rich in plant fiber [51,52]. This suggests that the more diverse and abundant intake of plant-based foods in natural environments may promote the proliferation of these bacteria. In contrast, the CB group demonstrated the enrichment of genera such as *Hallerella*, *Bullifex*, and *Treponema_D* at the genus level, and specific species such as *Hallerella intestinalis*, *Prevotella populations*, and *Treponema_D berlinense* at the species level. *Treponema* enrichment may be associated with inflammation [53,54], whereas the functional roles of other microbial taxa remain incompletely characterized. These observed enrichments likely stem from the relatively stable dietary composition and enclosed rearing environments of captive animals [55].

Several essential functions conferred by the gut microbiome on the host testify to its importance [56]. These include the fermentation of indigestible food components into absorbable metabolites, the synthesis of essential vitamins, the removal of toxic compounds, competition with pathogens, the strengthening of the intestinal barrier, and the stimulation and regulation of the immune system [57,58]. We conducted a KEGG pathway enrichment analysis to assess the functional profiles of fecal microbiota from ring-tailed lemurs in the CB and FR groups [59]. The results revealed significant differences between the two groups in pathways related to energy metabolism and carbon utilization [60,61] (oxidative phosphorylation, the citrate cycle, starch and sucrose metabolism, and pentose and glucuronate interconversions), nucleotide metabolism and cell proliferation [62] (urine metabolism, nucleotide metabolism, pyrimidine metabolism, biosynthesis of nucleotide sugars, and biosynthesis of various nucleotide sugars), as well as microbial competition and defense mechanisms [63] (streptomycin biosynthesis, the bacterial secretion system, and protein export). Furthermore, we analyzed the differential genes in the oxidative phosphorylation pathway. In the FR group, several key genes (those associated with complexes I, II, and cytochrome oxidase) exhibited enhanced expression, suggesting that under free-ranging conditions, the microbiota elevate their energy acquisition and regulatory capacities to cope with more variable nutritional and environmental conditions [64]. Under captive conditions, where both the diet and the environment are relatively uniform, the abundance of certain electron transport chain components (such as nuoF, nuoE, and ahaH) increased, presumably as an adaptation to a stable and singular carbon source supply. The relative differences in gene expression within the streptomycin biosynthesis pathway between the two groups further imply that under natural free-ranging conditions, the microbiota may tend to produce greater amounts of secondary metabolites associated with antibiotic synthesis, thereby regulating community structure and maintaining ecological balance.

The colonic microbiome encodes many CAZymes to degrade carbohydrates beyond the capabilities of the host [65]. In our study, the composition and abundance of CAZyme genes in the fecal microbiota of ring-tailed lemurs differed significantly between the two rearing systems. Notably, in the FR group, the enzyme genes that were significantly elevated were primarily associated with the degradation of complex polysaccharides (cellulose, hemicellulose, and pectin), suggesting that semi-free-ranging lemurs may ingest more foods rich in plant fibers and complex carbohydrates (such as wild fruits, leaves, and bark). This dietary pattern likely stimulates the gut microbiota to secrete a variety of enzymes to fully utilize these complex carbohydrates, thereby providing essential energy and nutrients to the host. In contrast, the CB group predominantly harbored enzyme genes related to the degradation of starch, oligosaccharides, and simple carbohydrates. These findings are corroborated by our previous results on microbial diversity, metabolic pathways, and energy metabolism patterns. Similar trends have been observed in other captive primates. For example, howler monkeys (*Alouatta* spp.) and chimpanzees (*Pan troglodytes*) in captivity show marked reductions in fiber-degrading bacterial taxa, including *Prevotella* and *Ruminococcus*, likely due to a lower dietary fiber intake and reduced environmental microbial exposure [43,66]. These convergent patterns across species suggest a broader ecological principle: captivity often leads to a decline in microbial diversity and the loss of environmentally acquired, functionally important taxa, especially those involved in complex carbohydrate degradation [67].

The global production and utilization of antibiotics have become increasingly widespread, accompanied by an alarming escalation in antibiotic resistance. Infections caused by drug-resistant pathogens pose significant threats to human health and impose considerable burdens on the global economy [68]. The presence of antibiotic resistance genes (ARGs) underlies the origin and molecular basis of bacterial resistance. These ARGs and antibiotic-resistant bacteria are ubiquitously present in soil and freshwater environments [69,70]. In our study, the free-ranging group of ring-tailed lemurs exhibited a higher abundance of ARGs compared to the captive group, which corroborates this perspective. Previous studies have evaluated the risks associated with antibiotics by comprehensively considering factors such as human-associated enrichment, gene mobility, and host pathogenicity [71]. Among these, Type I ARGs have been classified as high-risk resistance genes (those that are enriched in human-associated environments, exhibit gene mobility, and are harbored by pathogenic bacteria), whereas Type II ARGs refer to those not yet present in human pathogens but carrying a high risk of transfer to human pathogens. Notably, when comparing the antibiotic risk profiles in the fecal microbiota of the two groups of ring-tailed lemurs, the CB group exhibited a higher abundance of Type I ARGs than the FR group, while the FR group showed significantly higher levels of Type II and Type III ARGs compared to the CB group. This indicates that captive environments may facilitate the enrichment of high-risk ARGs, whereas free-ranging environments may be associated with greater exposure to low-risk ARGs.

In this study, we employed a shotgun metagenomic approach to characterize the taxonomic composition and functional potential of the gut microbiome in ring-tailed lemurs (*Lemur catta*) maintained under two distinct rearing regimes. By systematically comparing captive and semi-free-range individuals, we substantially expanded the dataset on primate microbiome responses to environmental variation, thereby facilitating cross-species meta-analyses. Our results demonstrate that captivity induces a pronounced restructuring of the lemur gut community, marked by a significant reduction in bacterial α-diversity and the selective enrichment of taxa associated with high-carbohydrate diets. Such microbial shifts may compromise host metabolic flexibility, underscoring the need to reformulate captive diets—particularly through the inclusion of additional dietary fiber—to better mimic natural foraging substrates. Nonetheless, this investigation has several limitations. First, although our sample size (n = 14 per group) was determined via a priori power analysis, it may still constrain the extrapolation of findings to more genetically and ecologically diverse populations. Second, all subjects originated from a single facility in Shanghai, China, limiting the geographic and environmental breadth of inference. Future work should aim to incorporate larger cohorts across multiple locales and husbandry conditions to validate and extend the environmental–microbiome–host interactions identified here.

## 5. Conclusions

In summary, the results show that captivity altered the community structure of the fecal microbiota in these animals. Differences in the living environment resulted in variations in microbial functions and in the profiles of carbohydrate-active enzyme genes. Furthermore, the microbial community composition among captive ring-tailed lemurs was more homogeneous, yet it harbored a higher abundance of potential pathogens, suggesting that a captive lifestyle may adversely affect gastrointestinal health.

## Data Availability

The original contributions presented in the study are included in the article; further inquiries can be directed to the author.
